# Improved reproducibility of diffusion tensor image analysis along the perivascular space (DTI-ALPS) index: an analysis of reorientation technique of the OASIS-3 dataset

**DOI:** 10.1007/s11604-022-01370-2

**Published:** 2022-12-06

**Authors:** Hiroyuki Tatekawa, Shu Matsushita, Daiju Ueda, Hirotaka Takita, Daisuke Horiuchi, Natsuko Atsukawa, Yuka Morishita, Taro Tsukamoto, Taro Shimono, Yukio Miki

**Affiliations:** 1 Department of Diagnostic and Interventional Radiology, Graduate School of Medicine, Osaka Metropolitan University, 1-4-3, Asahi-machi, Abeno-ku, Osaka, 545-8585 Japan; 2 Smart Life Science Lab, Center for Health Science Innovation, Osaka Metropolitan University, Osaka, Japan

**Keywords:** ALPS index, Reorientation, Glymphatic system, Reproducibility

## Abstract

**Purpose:**

Diffusion tensor image analysis along the perivascular space (DTI-ALPS) index is intended to reflect the glymphatic function of the brain; however, head rotation may reduce reproducibility and reliability. This study aimed to evaluate whether reorientation of DTI data improves the reproducibility of the ALPS index using the OASIS-3 dataset.

**Materials and methods:**

234 cognitively normal subjects from the OASIS-3 dataset were included. Original and reoriented ALPS indices were calculated using a technique that registered vector information of DTI to another space and created reoriented diffusivity maps. The *F* test was used to compare variances of the original and reoriented ALPS indices. Subsequently, subjects with head rotation around the *z*- (inferior-superior; *n* = 43) or *x* axis (right-left; *n* = 25) and matched subjects with neutral head position were selected for evaluation of intra- and inter-rater reliability. Intraclass correlation coefficients (ICCs) of the original and reoriented ALPS indices for participants with head rotation and neutral head position were calculated separately. The Bland–Altman plot comparing the original and reoriented ALPS indices was also evaluated.

**Results:**

The reoriented ALPS index exhibited a significantly smaller variance than the original ALPS index (*p* < 0.001). For intra- and inter-reliability, the reorientation technique showed good-to-excellent reproducibility in calculating the ALPS index even in subjects with head rotation (ICCs of original ALPS index: 0.52–0.81; ICCs of reoriented ALPS index: > 0.85). A wider range of the 95% limit of agreement of the Bland–Altman plot for subjects with *x* axis rotation was identified, indicating that *x* axis rotation may remarkably affect calculation of the ALPS index.

**Conclusion:**

The technique used in this study enabled the creation of reoriented diffusivity maps and improved reproducibility in calculating the ALPS index.

**Supplementary Information:**

The online version contains supplementary material available at 10.1007/s11604-022-01370-2.

## Introduction

Dysfunction of the glymphatic system has been revealed to be associated with various diseases, including neurodegenerative disorders such as Alzheimer’s [1, 2] and Parkinson’s disease [3], as well as sleep and mental disorders [4]. An accurate evaluation of the glymphatic system is required to predict the degree or progression of such diseases.

Diffusion tensor image analysis along the perivascular space (DTI-ALPS) was recently suggested and estimated to reflect the glymphatic function or interstitial fluid dynamics of the brain [5]. The ALPS index has been evaluated for several diseases and is expected to become a convenient and useful imaging biomarker because it can be easily calculated from the DTI sequence of magnetic resonance imaging (MRI) [6–8]. To calculate the ALPS index, the regions of interest (ROIs) were placed on the projection and association fibers adjacent to the medullary veins at the level of the lateral ventricle, and then applied to the diffusivity maps in the direction of the *x*- (right-left), *y*- (anterior–posterior), and *z*- (inferior-superior) axes. To obtain an accurate and highly reproducible ALPS index, uniform ROI placement and a rigid head position during MR examination are required. Some previous techniques were suggested to automatically place ROIs because automated techniques can provide a highly reproducible ALPS index [9, 10]. Meanwhile, Taoka et al. [11] reported a reduced ALPS index with head rotation, especially in a chin-raising position, compared with that obtained from the same participants with a neutral head position. Automated ROI placement might also not be successful when head rotation takes place. Hence, subjects whose head position or imaging plane has severely rotated during the MR examination should be excluded from studies, because their ALPS index is unreliable. To avoid wasting resources due to inappropriate head position, it is important to validate the reliability of the reorientation techniques by evaluating the reproducibility of the ALPS index for such subjects.

In the current study, we used a technique that registered vector images to another space and created a reoriented diffusivity map. This technique may be used to easily calculate the ALPS index with high reproducibility, even for subjects with inappropriate head position or imaging plane during DTI acquisition. Therefore, this study aimed to evaluate whether the DTI reorientation technique improved reproducibility of the ALPS index using a large open-source dataset.

## Materials and methods

### Subjects

All data were downloaded from the OASIS-3 brain project (http://oasis-brains.org/) [12], which is a freely available neuroimaging dataset for subjects with normal aging and Alzheimer’s disease containing 2842 MR sessions of 1379 subjects. Inclusion criteria were subjects who were cognitively normal and had DTI data available with 64 directions of motion probing gradient (MPG) and a *b* value of 1000 s/mm^2^. For the 23 subjects who underwent MR examination including DTI sequence twice, the first DTI datasets were used. In addition, the second DTI datasets were used for the subsequent test–retest study. Finally, 234 participants were selected. Clinical information, including age, sex, dominant hand, and Mini-Mental State Examination results, was retrieved from the Alzheimer Disease Research Center data of the date closest to that of the MR examination. Since this dataset is open-source, written informed consent of participants, ethics board review and approval, and conformance to the Declaration of Helsinki were not required.

### MRI preprocessing

All MRI data from the OASIS-3 database were acquired using a 3 T scanner (Biograph mMR, Siemens, Erlangen, Germany). The parameters of the DTI comprised 64 directions of MPG with *b* value = 1000 s/mm^2^, along with a single *b* value = 0 s/mm^2^ image on anterior-to-posterior phase encoding: repetition time/echo time = 11,000/87 ms, flip angle = 90°, slice thickness = 2.5 mm without interslice gap, and matrix size = 96 × 96 (2.5 × 2.5 × 2.5 mm isovoxel). Detailed additional information is provided on the website (https://www.oasis-brains.org/files/OASIS-3_Imaging_Data_Dictionary_v2.0.pdf). Before subsequent analysis, all DTI were denoised, Gibbs ringing artifacts were removed, and the motion, eddy current, and bias fields were corrected to reduce undesired distortions or artifacts using MRtrix3 software (*dwidenoise, mrdegibbs, dwifslpreproc, dwibiascorrect function*; https://www.mrtrix.org) [13].

To calculate the original and reoriented ALPS (ro-ALPS) indices, two sets of maps derived from DTI were created using the FMRIB Software Library version 6.0 (FSL; Oxford, UK; www.fmrib.ox.ac.uk/fsl) and Analysis of Functional NeuroImages software (AFNI; NIMH Scientific and Statistical Computing Core; Bethesda, MD, USA; https://afni.nimh.nih.gov).

1. Original dataset: fractional anisotropy (FA) maps, color-coded maps, and diffusivity maps in the direction of the *x* axis (right-left; *Dx*), *y* axis (anterior–posterior; *Dy*), and *z* axis (inferior-superior; *Dz*) were obtained for each subject using FSL software (*dtifit function*).
2. Reoriented dataset: the FA map of the original dataset of all individuals was registered to the ICBM FA map (1 mm isovoxel) with six degrees of freedom rigid transformation using FSL software (*flirt function*), and the transformation matrices were calculated; translation and rotation, not scaling, were performed for the registration. Then, the vector data were registered using the transformation matrices to create a reoriented diffusivity map with reoriented *x* axis (reoriented right-left; *ro-Dx*), *y* axis (reoriented anterior–posterior; *ro-Dy*), and *z* axis (reoriented inferior-superior; *ro-Dz*) directions, as well as a reoriented color-coded map using the *vecreg function* of FSL that enables registration of vector data to another space (the ICBM template space in the current study).

**Fig. 1:**
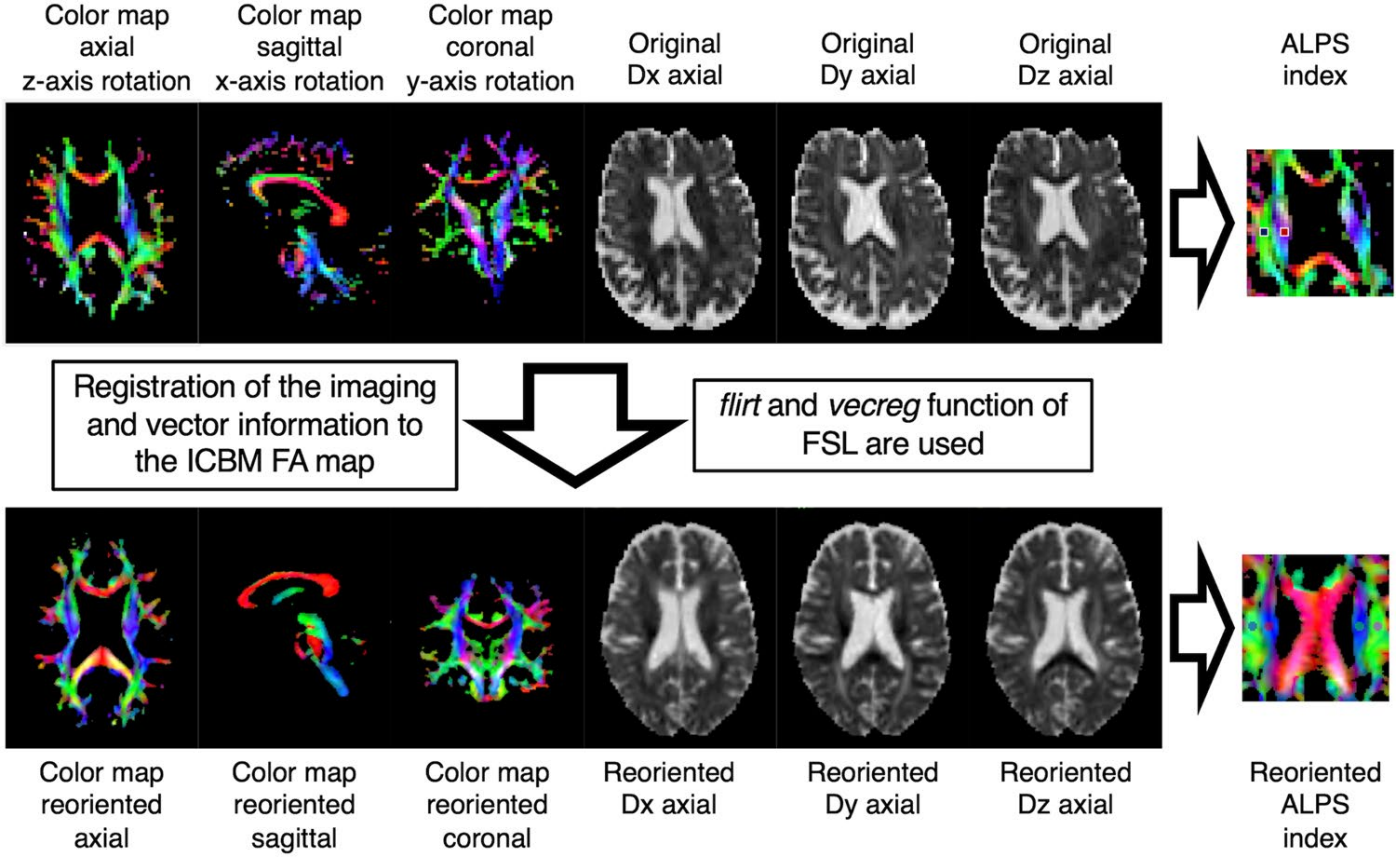
FA map with *x*-, *y*-, and *z* axes’ rotations is registered to ICBM FA map; color-coded map and diffusivity map as well as vector information are registered (*flirt* and *vecreg* function). Reoriented color-coded map and diffusivity maps (*Dx*, *Dy*, and *Dz*) are created for calculating reoriented ALPS index. *FA* fractional anisotropy, *ALPS* analysis along the perivascular space

The process is illustrated in Fig. 1, and the process code is recorded as Supplemental data. To make it easier to understand, the figure shows a representative subject with moderate *x*-, *y*-, and *z* axes’ rotations.

### Calculation of ALPS and ro-ALPS indices

Four 5 mm spherical ROIs (volume = 62.5 mm^3^ [four voxels] for the original images and 57 mm^3^ [57 voxels] for the reoriented images) were manually marked in the region of the projection and association fibers for both hemispheres adjacent to the medullary veins at the level of the lateral ventricle body on the original and reoriented color-coded maps by a neuroradiologist with 14 years of experience (H.T.) using ITK-snap software (www.itksnap.org). For some of the subjects who were used for the reproducibility analysis, ROIs were also placed by the neuroradiologist (H.T.) again 2 weeks after the first procedure and by another neuroradiologist (S.M. with 7 years of experience). The DTI-ALPS index measures the ratio of the mean of the bilateral *x* axis diffusivity in the area of projection fibers and bilateral *x* axis diffusivity in the area of association fibers to the mean of bilateral *y* axis diffusivity in the area of projection fibers and bilateral *z* axis diffusivity in the area of association fibers according to the following formula [5]:

$$ALPS \, \,index\, \, = \, \, mean\left( {D_{x,proj} , \, D_{x,assoc} } \right)/mean\left( {D_{y,proj} , \, D_{z,assoc} } \right)$$

$$ro - ALPS \, index \, = \, mean\left( {ro - D_{x,proj} , \, ro - D_{x,assoc} } \right)/mean\left( {ro - D_{y,proj} , \, ro - D_{z,assoc} } \right)$$

### Statistical analysis

The ALPS and ro-ALPS indices exhibited normal distribution, as evaluated by the Shapiro–Wilk test. The original ALPS and ro-ALPS indices were compared using the paired *t* test, and their variances were evaluated using the *F *test. A linear regression model was used to evaluate the association between age and ALPS or ro-ALPS index for different sexes.

**Fig. 2:**
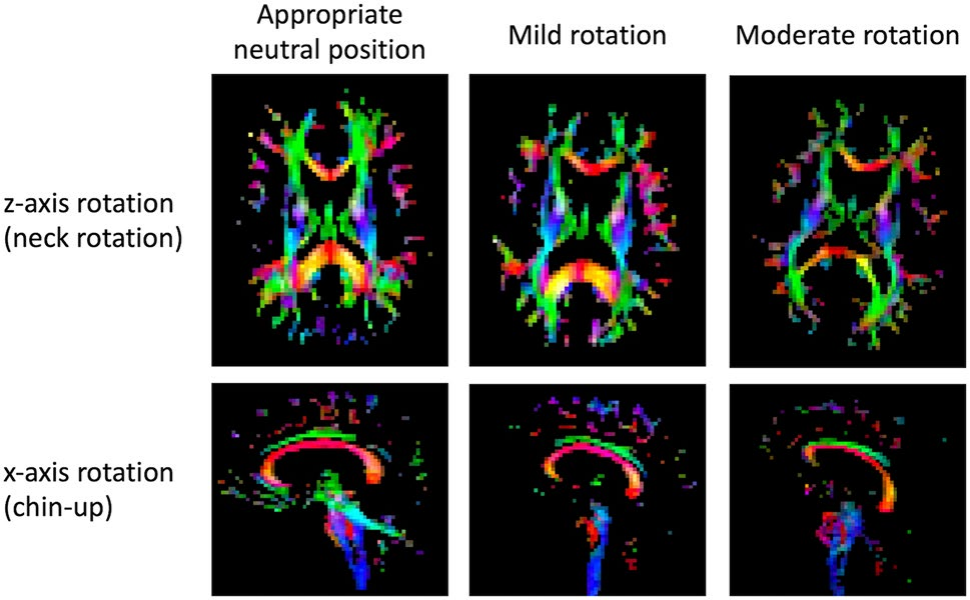
Examples of mild and moderate head rotation around the *z*- or *x* axis as well as of appropriate neutral position

Before evaluating the reproducibility of the ALPS index, two neuroradiologists (H.T. and S.M.) independently evaluated the degree of head rotation (0, appropriate neutral position; 1, mild rotation; 2, moderate rotation) on the axial (*z* axis rotation; neck rotation) and sagittal (*x* axis rotation; chin-up or down based on the anterior commissure–posterior commissure line as reference) planes, and the weighted *κ* coefficient was calculated. An example schema is shown in Fig. 2. Then, subjects judged to show moderate head rotation by both raters and age- and sex-matched subjects judged with an appropriate head position by both raters were included in the subsequent reproducibility analyses. Since most cases with *y* axis rotation coexisted with the *x*- or *z* axis rotation, the *y* axis rotation was not grouped in this study. Intraclass correlation coefficients (ICCs) of the ALPS and ro-ALPS indices with different head rotations were calculated to separately evaluate the intra- and inter-rater reliability. The weighted *κ* coefficient was defined as poor agreement, ≤ 0.2; fair agreement, 0.2–0.4; moderate agreement, 0.4–0.6; good agreement, 0.6–0.8; and very good agreement, > 0.8. The ICC was categorized as poor reliability, ≤ 0.5; moderate reliability, 0.5–0.75; good reliability, 0.75–0.9; and excellent reliability, > 0.9. The Bland–Altman plot between the ALPS and ro-ALPS indices with different head rotations was also evaluated. In addition, in subjects who underwent DTI examination twice, test–retest evaluation between the ALPS index of the first and second examinations was performed using paired *t* test, ICC analysis, and the Bland–Altman plot.

*P* < 0.05 was considered statistically significant. Statistical analyses were performed using GraphPad Prism version 9.4.1 software (GraphPad Software, San Diego, CA, USA; https://www.graphpad.com/scientific-software/prism/).

## Results

234 participants (mean age ± standard deviation [SD], 71.4 ± 8.4 [range, 42–92] years; females, 111 [47%]; right-handed, 217 [93%]; mean Mini-Mental State Examination ± SD, 29.2 ± 1.1 [range 25–30]) were included in this study.

**Fig. 3:**
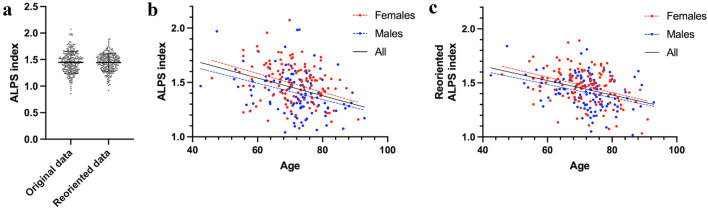
**a** The paired *t* test shows no significant difference, while the variance is significantly smaller for the ro-ALPS index than for the original ALPS index. **b** ALPS and **c** ro-ALPS indices are significantly associated with age for female, male, and combined groups. *ALPS* analysis along the perivascular space

When comparing the ALPS and ro-ALPS indices, the paired *t* test exhibited no significant difference (*p* = 0.80), whereas the variance was significantly smaller for the ro-ALPS index than for the original ALPS index (*F* = 2.40; *p* < 0.001, Fig. 3a). Both ALPS and ro-ALPS indices were significantly associated with age in the female, male, and combined groups (all *p* < 0.001, Fig. 3b, c).

For the evaluation of the intra- and inter-rater reliability of the ALPS index, 43 subjects with head rotation around the *z* axis evaluated on the axial plane, 25 subjects with head rotation around the *x* axis evaluated on the sagittal plane, and age- and sex-matched subjects with neutral head position were selected (evaluation of *z* axis rotation [*n* = 43]: weighted *κ* coefficient, 0.89; mean age ± SD, 73.8 ± 7.1 vs. 73.2 ± 6.6 years; females, 15 [35%], right-handed, 42 [98%]; evaluation of *x* axis rotation [*n* = 25]: weighted *κ* coefficient, 0.71; mean age ± SD, 75.0 ± 7.6 vs. 74.7 ± 7.5 years; females, 19 [76%], right-handed, 25 [100%]). Five subjects had *z*- and *x* axes head rotation.

The intra- and inter-rater ICC values are presented in Table 1. For the intra-rater ICC evaluation, the ICCs of the original ALPS index were 0.55–0.81, whereas those of the ro-ALPS were 0.92–0.97. The difference in head rotation status had little effect on the intra-rater reproducibility of the original and reoriented ALPS indices. For the inter-rater ICC evaluation, the ICCs of the original ALPS index were 0.52–0.67, while those of ro-ALPS were 0.85–0.94. The difference in head rotation also had little effect on the inter-rater reproducibility of the original and reoriented ALPS indices. In all reproducibility analyses, the ro-ALPS index exhibited better intra- and inter-rater ICCs than the original ALPS index.

**Table 1:** Intra- and inter-rater ICC with different head positions

	Intra-rater ICC		Inter-rater ICC	
	ALPS index	Reoriented ALPS index	ALPS index	Reoriented ALPS index
Subjects with *z* axis rotation (*n* = 43)	0.55	0.93	0.66	0.94
Subjects with neutral position (*n* = 43)	0.66	0.96	0.64	0.91
Subjects with *x* axis rotation (*n* = 25)	0.81	0.97	0.67	0.92
Subjects with neutral position (*n* = 25)	0.62	0.92	0.52	0.85

*ICC* intraclass correlation coefficient, *ALPS* analysis along the perivascular space

**Fig. 4:**
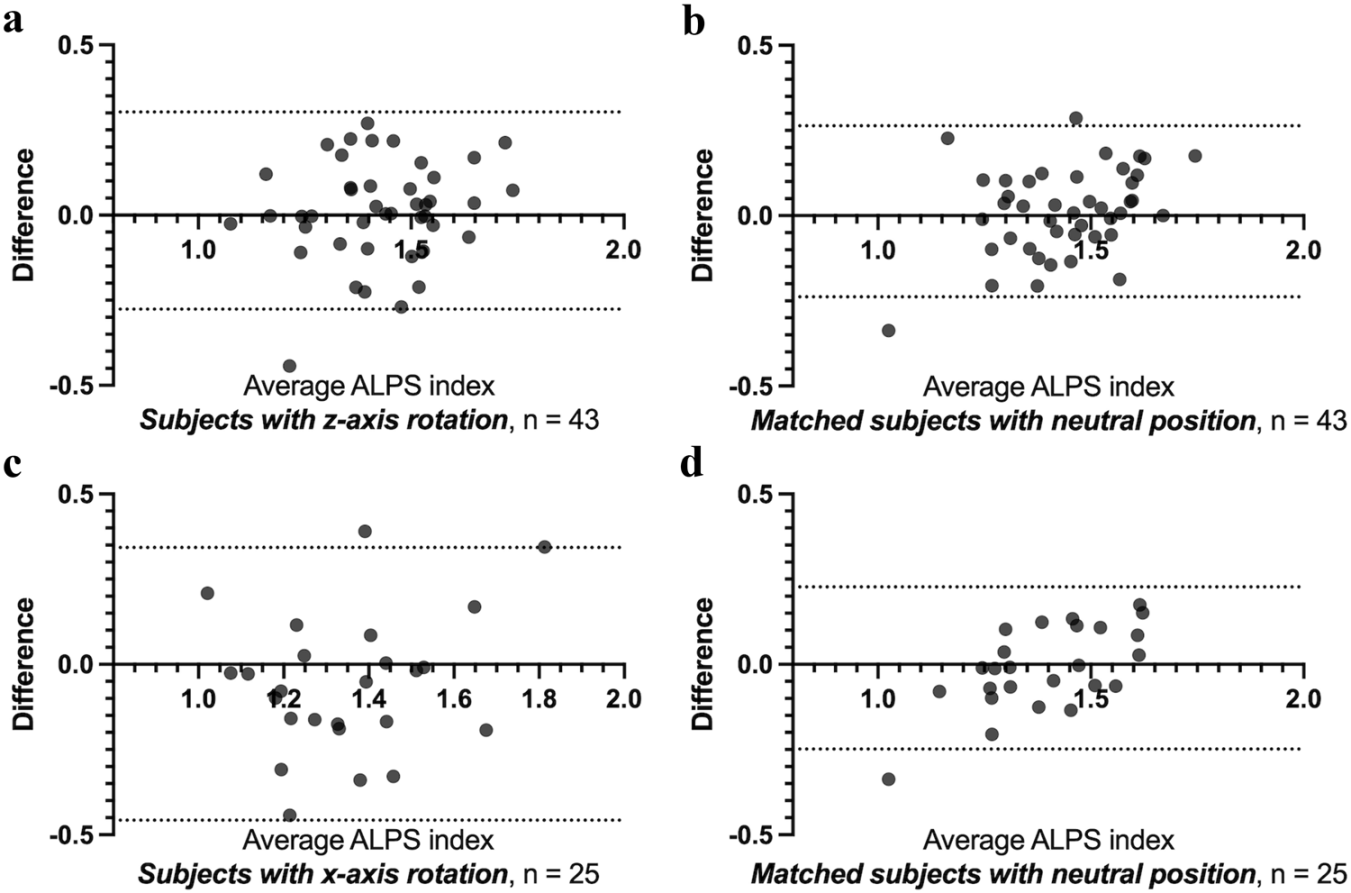
Bland–Altman plot comparing the ALPS and ro-ALPS indices. A similar range of the 95% limit of agreement between the subjects with **a** *z* axis head rotation and **b** neutral head position is found. A wider range of the 95% limit of agreement in **c** subjects with *x* axis head rotation compared with **d** that of subjects with neutral head position is identified. The *x* axis indicates the mean value of the ALPS and ro-ALPS indices, whereas the *y* axis indicates the difference between the ALPS and ro-ALPS indices. Two dotted lines indicate the 95% limit of agreement. *ALPS* analysis along the perivascular space

In the Bland–Altman plot comparing the ALPS and ro-ALPS indices (Fig. 4), subjects with *z* axis rotation displayed a similar 95% limit of agreement compared with subjects with an appropriate neutral head position (− 0.27 to 0.30 vs. − 0.24 to 0.26), while subjects with *x* axis rotation exhibited a wider 95% limit of agreement compared with subjects with a neutral head position (− 0.45 to 0.34 vs. − 0.25 to 0.22). In both head rotations, the paired *t* test did not demonstrate significant differences between the ALPS and ro-ALPS indices (all *p* > 0.17).

Every ALPS index and mean value of *mean(Dx,proj, Dx,assoc)* and *mean(Dy,proj, Dz,assoc)* used for reproducibility evaluation are shown in Supplemental Fig. 1 and Supplemental Table 1, respectively.

**Fig. 5:**
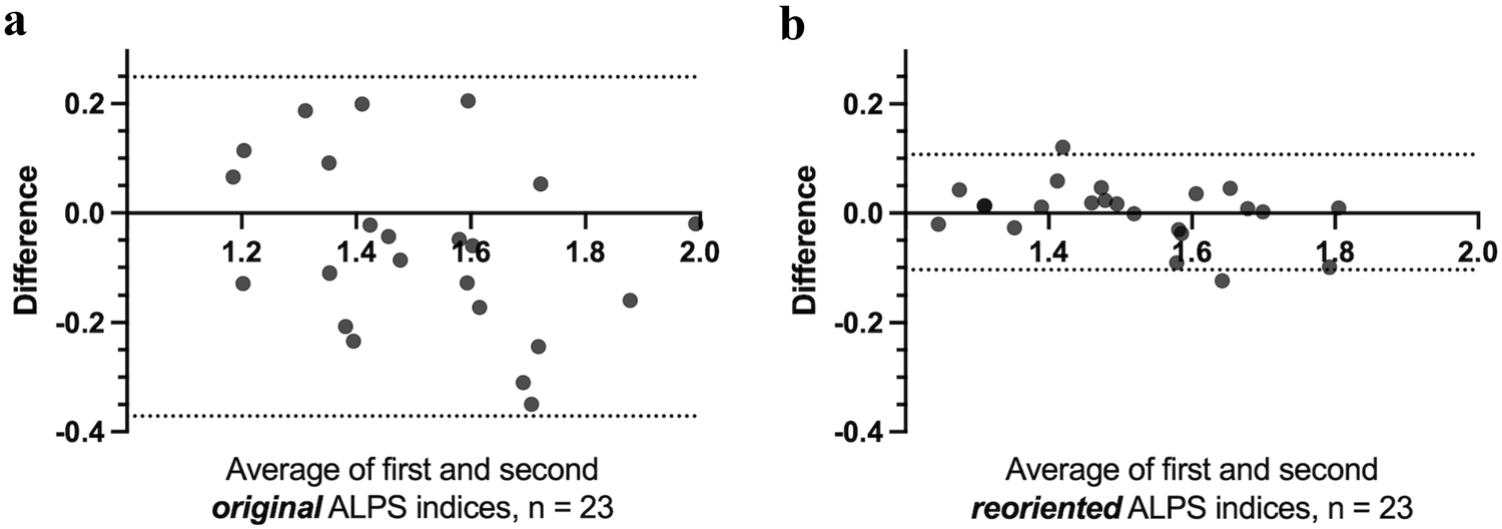
Bland–Altman plot comparing the first and second ALPS or ro-ALPS indices. The original ALPS index exhibited a wider 95% limit of agreement. Two dotted lines indicate the 95% limit of agreement. *ALPS* analysis along the perivascular space

For the test–retest evaluation, data from 23 subjects (mean interval date between the MR examinations, 724 days [range 14–1136 days]) were analyzed to compare between the first and second ALPS indices. When comparing between the first and second ALPS indices of original or reoriented data, there were no significant differences (paired *t* test: original ALPS index, *p* = 0.08; reoriented ALPS index, *p* = 0.89). The ICC between the first and second original ALPS indices was 0.74, whereas the ICC between the first and second reoriented ALPS indices was 0.95. In the Bland–Altman plot comparing the first and second ALPS indices of original or reoriented data (Fig. 5), the original ALPS index exhibited a wider 95% limit of agreement.

## Discussion

The ro-ALPS index exhibited a significantly smaller variance than the original ALPS index. In the evaluation of intra- and inter-reliability, the reorientation technique showed good-to-excellent reproducibility in calculating the ALPS index even in subjects with head rotation. The wider range of the 95% limit of agreement of the Bland–Altman plot for subjects with *x* axis rotation indicated that *x* axis rotation may considerably affect the calculation of the original ALPS index.

Compared with conventional MR sequences, DTI data are complicated because they contain both signal intensity information and vector information [14]; therefore, DTI analyses, especially tractography and connectome evaluation, may be recommended to be performed on the original DTI space. In contrast, this study calculated the ALPS index on the reoriented space. One reason is that the ALPS index is a simple metric and is not influenced by continuity of the voxels. The vector directions along the *x*-, *y*-, and *z* axes in relation to the brain are crucial for accurate calculation of the ALPS index. A previous study also reported that alterations in the imaging plane and head position largely influenced reproducibility of the ALPS index [11], and that it may be appropriate to determine the imaging plane according to the direction of the anterior commissure–posterior commissure line. The technique used in this study enabled easy creation of diffusivity maps with the reoriented *x*-, *y*-, and *z* axes, and the imaging plane of the reoriented diffusivity maps appeared to show an appropriate direction in relation to the head position. Hence, the reoriented head position and fitted diffusivity maps may lead to a smaller variance in the ALPS index.

For reproducibility analyses, the ro-ALPS index showed good-to-excellent intra- and inter-rater reliability, even when the head position was moderately rotated. Using the reorientation technique, the best regions for ROI placement at the projection and association fibers can be easily identified, leading to very good intra- and inter-rater reliabilities. In this study, we manually placed ROIs to evaluate the reproducibility of this technique, whereas previous studies introduced other methods to automatically or semi-automatically place ROIs [9–11, 15]. A combination of these methods may further improve the reproducibility of calculating the ALPS index, although our method has already demonstrated good-to-excellent intra- and inter-reliability. Another advantage of our technique is that a reoriented diffusivity map, which corrects the *x*-, *y*-, and *z* directions of diffusivity fitted to each brain, can be created. Zhang et al. [10] registered the original diffusivity map to the brain template, but did not register vector information; hence, their technique could not be applied to subjects with head rotation. The FSL function “*vecreg*” is a command-line tool that allows the registration of vector data. It does not calculate a transformation but simply applies a given transformation to the input vector field. This function can be applied to the V1 (1st eigenvector), V2 (2nd eigenvector), V3 (3rd eigenvector), and diffusivity maps. Therefore, this technique may possess sufficient function for calculating the ro-ALPS index. When the original DTI with multiple MPG is registered to a target image and the reoriented *b*-vector information could be calculated using other methods, reoriented diffusivity maps could also be created; however, this might be complicated. In contrast, our technique could create reoriented diffusivity maps using only the FSL function; therefore, this technique could be easily used to calculate the ALPS index even when DTI data were not obtained with an appropriate head position. To our knowledge, this technique has been used in a few recent studies [16, 17]; however, reproducibility of the ALPS index has not been evaluated. We believe this technique would be useful for improving intra- and inter-reliability and will be beneficial in future studies to estimate glymphatic function of the brain.

The Bland–Altman plot between the ALPS and ro-ALPS showed a wider range of the 95% limit of agreement for subjects with *x* axis rotation (all cases with chin-up) than for subjects with an appropriate head position, indicating that *x* axis rotation may considerably affect the calculation of the original ALPS index. This finding was in agreement with that of a previous study that reported a relatively lower ALPS index for subjects with chin-up [11]. As shown in Supplemental Table 1, although subjects with *x* axis head rotation tended to show high *mean(Dx,proj, Dx,assoc)* and high *mean(Dy,proj, Dz,assoc)*, the high *mean(Dy,proj, Dz,assoc)* may have derived in relatively lower values for the ALPS index. Meanwhile, *z* axis rotation (neck rotation) may have little effect on the calculation of the original ALPS index, partly because this study placed ROIs on both hemispheres and included subjects with neck rotations to both sides. Nonetheless, alteration in the head position and imaging plane had a remarkable impact on the calculation of the ALPS index.

Although the sample size was not large, the test–retest evaluation confirmed that the reorientation technique shows high reproducibility for the calculation of the ALPS index.

The major limitation of this study was that we used open-source DTI data, and that most DTI datasets were obtained once, with a single head position. The DTI data of the OASIS-3 were obtained with consistent acquisition parameters and the same number of MPG directions. When we evaluated the correlation between the ALPS index and age, a significant association was identified, which was compatible with the results of previous studies [10, 11, 18, 19]. Hence, the OASIS-3 data seemed reliable to some extent. To determine whether the ro-ALPS index is truly reliable, further validation is required using a large sample of subjects who underwent MR examination with a rigid neutral head position and with a rotated head position. In contrast, the current study would be a good simulation of retrospective studies because the head position could not be corrected for previously obtained DTI data. Second, the imaging resolution and size of the ROIs used in the calculation of the original and reoriented ALPS indices were different, which may have affected their values. In contrast, a mean value within the ROIs was mathematically used for calculating the ALPS index, which may have mitigated the effect of the difference in resolution or size. Third, although this study evaluated the degree of head rotation by visual inspection, it may be appropriate to decide the severity of head rotation by an objective method based on the transform matrix or the angle of the imaging plane in relation to the anterior commissure–posterior commissure line. However, the weighted κ coefficient of the severity of head rotation showed good agreement, which suggests that our method was acceptable to certain degree.

In conclusion, the registration technique in this study enabled creation of reoriented diffusivity maps along the *x*-, *y*-, and *z* axes in relation to the reoriented head position and improved reproducibility in calculating the DTI-ALPS index even when the head position or imaging plane was inappropriate. This technique has potential to be widely applied in future prospective and retrospective studies to evaluate the DTI-ALPS index.

## Supplementary Information

Below is the link to the electronic supplementary material.

Supplementary file1 (PDF 306 KB)

Supplementary file2 (DOCX 4 KB)

## Data Availability

The data that support the findings of this study are available from the corresponding author, [H.T.], upon reasonable request.
