# Supplementary material for: Improved reproducibility of diffusion tensor image analysis along the perivascular space (DTI-ALPS) index: an analysis of reorientation technique of the OASIS-3 dataset
Source: Jpn J Radiol. 2022 Dec 6;41(4):393–400. doi: 10.1007/s11604-022-01370-2 (PMC10066136; doi:10.1007/s11604-022-01370-2)
Supplement: Supplementary file 1 — Supplementary file1 (PDF 306 KB) [file 11604_2022_1370_MOESM1_ESM.pdf]

Supplemental Fig. 1

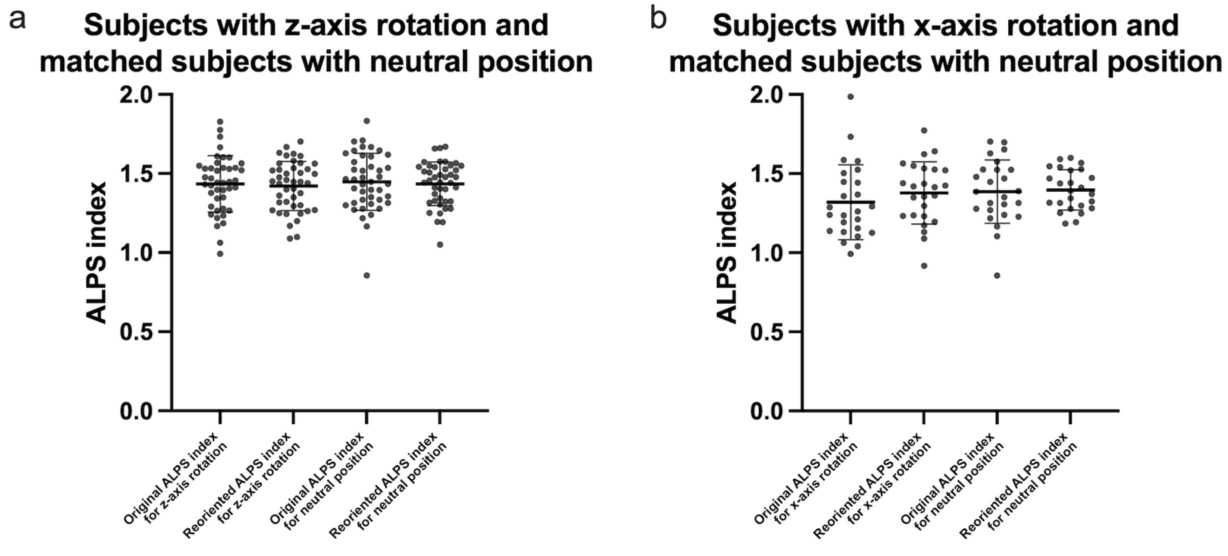

Supplemental Fig. 1 ALPS index used for reproducibility evaluation. Bars show the mean value and standard deviation. ALPS, analysis along the perivascular space

Supplemental Table 1 Mean values of *mean(Dx,proj, Dx,assoc)* and *mean(Dy,proj, Dz,assoc)*

	Original data		Reoriented data	
	mean(Dx,proj, Dx,assoc)	*mean(Dy,proj, Dz,assoc)*	*mean(Dx,proj, Dx,assoc)*	*mean(Dy,proj, Dz,assoc)*
Subjects with z-axis rotation (n = 43)	6.04×10^{-4}	4.25×10^{-4}	5.95×10^{-4}	4.21×10^{-4}
Subjects with neutral position (n = 43)	6.02×10^{-4}	4.22×10^{-4}	5.97×10^{-4}	4.18×10^{-4}
Subjects with x-axis rotation (n = 25)	6.41×10^{-4}	4.98×10^{-4}	5.93×10^{-4}	4.37×10^{-4}
Subjects with neutral position (n = 25)	5.98×10^{-4}	4.41×10^{-4}	5.96×10^{-4}	4.29×10^{-4}
